# Race/ethnicity, and Americans’ perceptions and experiences of over- and under-use of care: a cross-sectional study

**DOI:** 10.1186/s12913-015-1106-7

**Published:** 2015-10-01

**Authors:** Nancy R. Kressin, Meng-Yun Lin

**Affiliations:** Section of General Internal Medicine, Boston University School of Medicine, 801 Massachusetts Avenue, Crosstown Centre, Boston, MA 02118 USA; VA Boston Healthcare System, Boston, MA 02130 USA; Health Policy and Management Department, Boston University School of Public Health, Boston, MA 02118 USA

**Keywords:** Patient preferences, Disparities, Survey research

## Abstract

**Background:**

Despite widespread documentation of racial/ethnic disparities in care (predominantly under-use of needed care), differences in population-wide attitudes or experiences about under- or overuse (care where harms may outweigh potential benefits) of care are not well understood. We examined whether race/ethnicity is associated with perceptions or experiences of overuse or underuse.

**Methods:**

We conducted secondary analysis of a cross-sectional national telephone survey of nationally representative sample of 1238 American adults; 57.9 % female, 75.4 % Non-Hispanic White, 11.8 % Non-Hispanic Black, 10.1 % Hispanic. The main outcome measures are general perceptions and personal experiences of overuse and underuse, including cost-related dimensions of each.

**Results:**

Bivariate results indicated that respondents of minority race/ethnicity generally viewed both overuse and underuse as bigger problems than did Whites, and reported more personal experiences of each. After adjustment, Hispanics were less likely than Whites to report personal experiences of overuse (odds ratio [OR] [95 % CI], 0.44 [0.23 to 0.86]), while Blacks and Others were more likely to report cost-related overuse (ORs [95 % CIs], 4.16 [2.30 to 7.51]; 3.55 [1.52 to 8.28], respectively). Non-Hispanic Others more often reported doctors’ protection from overuse (OR [95 % CI], 3.69 [1.75 to 7.78]). General concerns with underuse were more frequent among Blacks and Hispanics (ORs [95 % CIs], 3.07 [1.72 to 5.54]; 2.12 [1.24 to 3.61] respectively), while Others reported significantly fewer concerns (OR [95 % CI], 0.43 [0.23 to 0.80]).

**Conclusions:**

Over- and underuse of medical care are important problems for many Americans, and experiences vary by race/ethnicity. Clinician communication and educational campaigns about appropriateness of care may need tailoring for varying population groups.

## Background

Racial/ethnic disparities in receipt of health care are widely documented [1, 2], but most research has focused on *under*use – patients not receiving needed and appropriate care — without also examining *over*use—receipt of unneeded or inappropriate care that does not improve health outcomes, whose harms and risks exceed its benefits [3–5]. The soaring U.S. health spending and suboptimal outcomes have put pressure on the US health care system to reduce costs and improve the quality of health care. Today, there are multiple efforts underway to increase awareness of the issue of overuse, as the elimination of unnecessary medical care may reduce waste and prevent potential harm. Most prior disparities studies have examined actual receipt of care among clinically similar patients, so little is known about attitudes or experiences about under- or over-use among the general American population. Such attitudes and perceptions are important to understand, as they might be a target for future interventions to improve the appropriateness of care.

Despite the increasing concerns regarding overuse of care, as exemplified by the American Board of Internal Medicine Foundation’s “Choosing Wisely” campaign, efforts by Consumer Reports, and others [4, 6, 7], and longstanding concerns about underuse [1], we could not locate any published reports of population-based studies of attitudes or related experiences with over- or underuse of health care, or how these vary by race. Thus, the goal of this study was to examine racial/ethnic differences in Americans’ attitudes and experiences about overuse and underuse of medical care. We hypothesized, given known disparities in underuse of care, that racial/ethnic minority respondents would be more likely to report both general concerns and personal experiences with underuse. We posited that given Whites’ greater relative socioeconomic advantage [8] (that might foster use of more care, including more inappropriate care [9–12]), Whites might perceive and experience more overuse.

## Methods

### Overview

Using publicly available data from a nationally representative sample of American adults, we examined the extent to which race/ethnicity was associated with perceptions and experiences of overuse and underuse. We developed our hypotheses prior to inspection of the data. Institutional Review Board approval was obtained from the Boston University Institutional Review Board with waiver of consent.

### Data

We used cross-sectional national telephone survey data from “The Public and the Health Care Delivery System”, which examined numerous aspects of the public’s attitudes and experiences [13]. This effort was jointly sponsored by National Public Radio (NPR), the Kaiser Family Foundation (KFF) and the Harvard School of Public Health (HSPH). Survey fieldwork was done by telephone in March, 2009 by Social Science Research Solutions, of a nationally representative sample of 1238 randomly selected respondents ages 18 and over. Interviews were conducted in English and Spanish. In the data file, all groups were weighted to reflect their actual distribution in the nation.

We accessed the data in May, 2013 through the Roper Center for Public Opinion Research at the University of Connecticut’s iPOLL Databank, to conduct analyses to address different scientific questions than those originally examined or reported by the study’s sponsors. The Boston University Institutional Review Board determined that this study was exempt from human studies review.

### Study variables

#### Independent variable

Race/ethnicity was classified as non-Hispanic White (hereafter “White”), non-Hispanic Black (“Black”), Hispanic, and non-Hispanic Others (“Others”), obtained via respondent self-report.

#### Outcomes

We examined two domains of respondents’ self-reported outcomes: perceptions about and experiences with 1) overuse of care, 2) underuse of care.

Overuse was assessed with 4 questions: 1) **general overuse** (“Too many patients getting medical tests and treatments that they don’t really need… is a major problem, minor problem, or not a problem”; responses to the latter two categories combined, don’t know responses deleted (3 %)). 2) **personal experiences with overuse** (“In the past 2 years, do you think you have received a medical test or treatment that was probably NOT necessary?”; response options were yes/no/don’t know (latter deleted; 1 %)). 3) **cost-related overuse** (“In the past 2 years, do you think your doctor has ever recommended an expensive medical test or treatment for you when a less expensive alternative would work just as well?”; response options: yes/no/don’t know (latter deleted; 5 %)). 4) **experiences with physicians*protecting*them from overuse** (“In the past 2 years, has a doctor denied you a medical test or treatment that you wanted because they thought it was not medically necessary?”; yes/no, don’t know (1 %; deleted)).

Underuse was assessed with 3 questions: 1) **general underuse of care** (“Too many patients NOT getting the medical tests and treatments that they need is a major problem, minor problem, or not a problem”, responses to the latter two categories combined, don’t know responses deleted (2 %)). 2) **personal experiences with underuse** (“In the past two years, has there been a time when you did NOT receive a medical test or treatment when you needed it?”; response options were yes/no/don’t know (latter deleted; 0.5 %)). 3) **cost-related underuse** (“In the past two years, do you think your doctor has ever recommended a medical test or treatment for you that is not as effective as other treatments but is less expensive?”; yes/no, don’t know (4 %; deleted)).

#### Covariates

We assessed sociodemographic factors which might affect individuals’ perceptions or experiences of care, or which might modify the association between race and the outcomes, including age (categorized as 18–29, 30–49, 50–64, 65 and over), gender, education (grouped into attended high school, graduated high school, attended college, and graduated college), family income (all-source income before taxes in 2008, dichotomized as <$50,000 and $50,000+), self-reported health status (categories: excellent, very good, good, fair or poor (latter two combined)), and presence/absence of insurance coverage (‘insured’ category including both private and government insurance (e.g., Medicare, Medicaid)).

### Statistical analysis

We first conducted bivariate analyses to explore the associations of race and other sociodemographic factors with the outcomes. Significance of associations between dependent and independent variables was determined by Pearson chi-square statistics if responses were dichotomous or by Mantel-Haenszel chi-square statistics for ordinal responses. All selected covariates were significantly associated with one or more of the outcomes, so all were later included in multivariate analyses. We then performed multivariate logistic regression analyses to examine whether race was significantly associated with the outcomes, after adjusting for covariates. Questions with more than two response options were recoded as dichotomous, as indicated above. To address issues of nonresponse bias, sampling weights were developed using US Census data, to reflect respondents’ actual distribution in the nation, which were supplied with the dataset and used in the analyses. Statistical significance was estimated at the level of *p* <0.05. All analyses were performed using SAS, version 9.3.1.

## Results

Demographic characteristics of the 1238 respondents showed that the majority of respondents were White (75 %), with about 12 % Black and 10 % Hispanic, and 58 % female (Table 1). More than 90 % of respondents were aged ≥30. About two-thirds of the participants attended or graduated college. Half of the subjects reported total family income from all sources before taxes less than $50,000. Almost half of the respondents reported excellent/very good health, and 90 % had some form of health insurance.

**Table 1:** Demographic characteristics of the sample (*N* = 1238)

Characteristics	% of subjects
Race/Ethnicity	
Non-Hispanic White	74.3 %
Non-Hispanic Black	11.6 %
Hispanic	9.9 %
Non-Hispanic Others	2.7 %
N/A	1.5 %
Gender	
Male	43.9 %
Female	56.1 %
Age	
18–29	6.3 %
30–49	32.0 %
50–64	31.4 %
65+	30.1 %
N/A	0.2 %
Education	
Attended high school	11.1 %
Graduated high school	24.9 %
Attended college	22.3 %
Graduated college	41.5 %
N/A	0.2 %
Family income^a^	
<$50,000	44.1 %
$50,000+	44.4 %
N/A	11.5 %
Self-reported health status	
Excellent	16.8 %
Very good	32.5 %
Good	27.8 %
Fair/Good	22.5 %
N/A	0.4 %
Insurance status	
Uninsured	9.9 %
Insured	90.0 %
N/A	0.1 %

^a^Total family income from all sources before taxes in 2008

### Overuse of care

Overall, 50 % of respondents felt that general overuse was a major problem, 14 % had personal experiences of overuse, 8 % reported experiences of cost-related overuse, and 8.5 % reported experiences of physicians protecting them from overuse. Bivariate results (Table 2) showed that race/ethnicity was consistently associated with perceptions of both general and personal overuse of health care, with racial/ethnic minorities generally reporting greater concerns with general overuse than Whites; Blacks and Others were more likely than Whites to report personal experiences with overuse, while Hispanics were least likely to report personal overuse. All racial/ethnic minority groups were more likely than Whites to report cost-related overuse, as well as doctors protecting them from overuse. Most other sociodemographic characteristics were significantly associated with perceptions of doctors protecting them from overuse, such that all racial/ethnic minorities, women, younger respondents, those who had attended but not graduated from high school or college, with lower incomes or poorer health status were more likely to report such experiences than Others, but there was no clear pattern as to which other sociodemographic factors were associated with the three other overuse items.

**Table 2:** Bivariate associations between sociodemographic characteristics and dependent variables

	Overuse								Underuse					
	General overuse a problem		Personal experiences with overuse		Cost-related overuse		Experiences with physicians protecting against overuse		General underuse a problem		Personal experiences with underuse		Cost-related underuse	
	Major problem (%)	Minor/no problem (%)	Yes (%)	No (%)	Yes (%)	No (%)	Yes (%)	No (%)	Major problem (%)	Minor/no problem (%)	Yes (%)	No (%)	Yes (%)	No (%)
Overall distribution	50.0	50.0	13.9	86.1	8.0	92.0	8.5	91.5	66.4	33.6	11.1	88.9	8.5	91.5
Race/Ethnicity														
Non-Hispanic White	46.8	53.2	13.3	86.7	5.5	94.5	7.2	92.8	61.7	38.3	9.6	90.4	6.8	93.2
Non-Hispanic Black	56.9	43.1	15.8	84.2	19.5	80.5	11.2	88.8	84.4	15.6	16.7	83.3	15.4	84.6
Hispanic	62.3	37.7	10.6	89.4	9.9	90.1	11.4	88.6	80.3	19.7	11.4	88.6	11.5	88.5
Non-Hispanic Others	54.8	45.2	33.3	66.7	23.3	76.7	27.3	72.7	60.6	39.4	21.2	78.8	15.2	84.8
*p*-value	0.029		0.001		<.001		<.001		<.001		0.026		0.002	
Gender														
Male	50.7	49.3	14.0	86.0	8.0	92.0	6.5	93.5	59.9	40.1	8.0	92.0	8.1	91.9
Female	49.0	51.0	13.8	86.2	8.0	92.0	10.0	90.0	71.6	28.4	13.5	86.5	8.8	91.2
*p*-value	0.295		0.979		0.679		0.021		<.001		<.001		0.731	
Age														
18–29	53.8	46.2	25.3	74.7	10.4	89.6	14.1	85.9	77.9	22.1	24.4	75.6	7.8	92.2
30–49	46.1	53.9	14.5	85.5	9.6	90.4	9.7	90.3	66.5	33.5	14.3	85.7	10.8	89.2
50–64	50.1	49.9	14.0	86.0	7.8	92.2	9.9	90.1	69.6	30.4	12.1	87.9	10.0	90.0
65+	52.4	47.6	11.0	89.0	5.8	94.2	4.3	95.7	60.7	39.3	3.8	96.2	4.4	95.6
*p*-value	0.815		<.001		0.485		0.003		0.009		<.001		0.100	
Education														
Attended high school	65.1	34.9	14.2	85.8	12.3	87.7	10.4	89.6	70.2	29.8	15.4	84.6	9.2	90.8
Graduated high school	54.7	45.3	12.2	87.8	9.7	90.3	7.2	92.8	71.5	28.5	12.4	87.6	9.2	90.8
Attended college	47.6	52.4	15.8	84.2	7.7	92.3	11.6	88.4	69.5	30.5	13.1	86.9	10.6	89.4
Graduated college	44.2	55.8	14.0	86.0	6.1	93.9	7.1	92.9	60.8	39.2	8.1	91.9	6.8	93.2
*p*-value	<.001		0.063		0.293		0.001		0.004		0.004		0.058	
Family income														
<$50,000	54.8	45.2	13.6	86.4	10.0	90.0	9.8	90.2	73.5	26.5	13.5	86.5	10.5	89.5
$50,000+	46.2	53.8	15.1	84.9	5.7	94.3	7.0	93.0	60.5	39.5	7.7	92.3	6.4	93.6
*p*-value	0.032		0.211		0.003		0.012		<.001		<.001		<.001	
Self-reported health														
Excellent	53.2	46.8	13.6	86.4	6.4	93.6	3.9	96.1	54.7	45.3	5.8	94.2	3.5	96.5
Very good	46.3	53.7	10.8	89.2	4.7	95.3	7.5	92.5	62.8	37.2	7.8	92.3	7.8	92.2
Good	46.2	53.8	13.5	86.5	8.6	91.4	9.4	90.6	69.5	30.5	10.5	89.5	7.6	92.4
Fair/Poor	56.7	43.3	19.1	80.9	13.5	86.5	12.3	87.7	76.5	23.5	20.3	79.7	14.6	85.4
*p*-value	0.521		0.007		0.085		0.003		<.001		<.001		<.001	
Insurance status														
Uninsured	51.7	48.3	13.9	86.1	10.9	89.1	12.3	87.7	81.5	18.5	27.9	72.1	16.7	83.3
Insured	49.4	50.6	13.9	86.1	7.7	92.3	8.0	92.0	64.7	35.3	9.2	90.8	7.5	92.5
*p*-value	0.148		0.380		0.894		0.144		<.001		<.001		<.001	

After adjusting for covariates in multivariate regressions, race/ethnicity remained a significant predictor of 3 overuse items, but not perceptions of general overuse (Table 3). Hispanics were less than half as likely as Whites to report personal overuse (adjusted odds ratio [AOR] [95 % CI], 0.44 [0.23 to 0.86]). Blacks and Others were three to four times more likely to report cost-related overuse (AORs [95 % CIs], 4.16 [2.30 to 7.51] and 3.35 [1.52 to 8.28], respectively). Non-Hispanic Others were more than three times more likely to report physicians protecting them from overuse (AOR [95 % CI], 3.69 [1.75 to 7.78]).

**Table 3:** Multivariate analyses: overuse of care^a^

	General overuse (Modelling ‘yes, major problem)	Personal experience with overuse (Modelling ‘yes’)	Cost-related overuse (Modelling ‘yes’)	Physician protection from overuse (Modelling ‘yes’)
N	959	974	948	973
Race/Ethnicity				
Non-Hispanic White (REF)				
Non-Hispanic Black	1.22 (0.79, 1.88)	1.48 (0.87, 2.50)	4.16 (2.30, 7.51)	1.68 (0.88, 3.20)
Hispanic	1.39 (0.90, 2.16)	0.44 (0.23, 0.86)	1.88 (0.93, 3.79)	0.99 (0.50, 1.97)
Non-Hispanic Other	1.13 (0.62, 2.05)	1.20 (0.59, 2.46)	3.55 (1.52, 8.28)	3.69 (1.75, 7.78)
Gender				
Male (REF)				
Female	0.87 (0.67, 1.14)	1.09 (0.75, 1.56)	0.99 (0.62, 1.60)	1.60 (1.01, 2.54)
Age				
18–29 (REF)				
30–49	0.66 (0.45, 0.95)	0.45 (0.29, 0.72)	1.03 (0.55, 1.93)	0.49 (0.28, 0.86)
50–64	0.75 (0.49, 1.13)	0.31 (0.18, 0.54)	0.77 (0.36, 1.64)	0.41 (0.21, 0.79)
65+	0.74 (0.46, 1.19)	0.27 (0.14, 0.52)	0.66 (0.28, 1.58)	0.19 (0.08, 0.48)
Education				
Attended high school (REF)				
Graduated high school	0.80 (0.50, 1.26)	0.85 (0.46, 1.58)	1.01 (0.48, 2.11)	0.53 (0.26, 1.09)
Attended college	0.54 (0.33, 0.88)	1.49 (0.80, 2.79)	1.16 (0.52, 2.58)	1.05 (0.51, 2.15)
Graduated college	0.44 (0.26, 0.73)	1.06 (0.54, 2.09)	1.09 (0.46, 2.62)	0.78 (0.35, 1.74)
Family income^b^				
<$50k (REF)				
$50,000+	0.96 (0.69, 1.33)	0.92 (0.60, 1.41)	0.56 (0.31, 1.00)	0.78 (0.46, 1.31)
Self-reported health status				
Fair/Poor (REF)				
Excellent	1.17 (0.74, 1.85)	0.43 (0.23, 0.81)	0.82 (0.38, 1.77)	0.25 (0.10, 0.63)
Very good	0.89 (0.60, 1.31)	0.44 (0.26, 0.73)	0.53 (0.27, 1.07)	0.46 (0.24, 0.88)
Good	0.85 (0.57, 1.26)	0.57 (0.35, 0.95)	0.82 (0.43, 1.54)	0.99 (0.56, 1.78)
Insurance status				
Uninsured (REF)				
Insured	1.72 (1.13, 2.62)	1.64 (0.92, 2.92)	1.48 (0.74, 2.95)	1.41 (0.74, 2.69)

^a^Logistic regressions models controlled for sample weights; all models included adjusted for all covariates

^b^Total family income from all sources before taxes in 2008

**Table 4:** Multivariate analyses: underuse of care^a^

	General underuse (Modelling ‘yes, major problem)	Personal experiences with underuse (Modelling ‘yes’)	Cost-related underuse (Modelling ‘yes’)
N	968	978	955
Race/Ethnicity			
Non-Hispanic White (REF)			
Non-Hispanic Black	3.07 (1.72, 5.45)	1.16 (0.63, 2.11)	1.72 (0.89, 3.29)
Hispanic	2.12 (1.24, 3.61)	0.61 (0.33, 1.13)	0.97 (0.47, 2.02)
Non-Hispanic other	0.43 (0.23, 0.80)	1.17 (0.52, 2.63)	0.49 (0.15, 1.59)
Gender			
Male (REF)			
Female	1.46 (1.09, 1.95)	1.99 (1.30, 3.05)	1.10 (0.68, 1.79)
Age			
18–29 (REF)			
30–49	0.72 (0.47, 1.09)	0.74 (0.45, 1.21)	1.98 (0.99, 3.98)
50–64	0.78 (0.49, 1.24)	0.43 (0.24, 0.79)	1.56 (0.72, 3.38)
65+	0.53 (0.31, 0.88)	0.09 (0.03, 0.26)	0.67 (0.23, 1.92)
Education			
Attended high school REF)			
Graduated high school	1.49 (0.90, 2.47)	0.37 (0.20, 0.70)	1.49 (0.67, 3.29)
Attended college	1.87 (1.08, 3.22)	1.09 (0.58, 2.04)	3.16 (1.38, 7.21)
Graduated college	1.56 (0.90, 2.71)	0.77 (0.38, 1.57)	2.32 (0.94, 5.74)
Family income^b^			
<$50k (REF)			
$50,000+	0.73 (0.51, 1.04)	0.50 (0.30, 0.82)	0.61 (0.34, 1.10)
Self-reported health status			
Fair/Poor (REF)			
Excellent	0.47 (0.29, 0.78)	0.16 (0.07, 0.35)	0.17 (0.06, 0.45)
Very good	0.85 (0.55, 1.31)	0.30 (0.17, 0.52)	0.29 (0.15, 0.55)
Good	0.93 (0.60, 1.44)	0.37 (0.21, 0.64)	0.37 (0.20, 0.70)
Insurance status			
Uninsured (REF)			
Insured	0.62 (0.38, 1.03)	0.87 (0.51, 1.47)	0.52 (0.28, 0.97)

Reference groups: NH-White, Male, Age: 18–29, Education: attended high school, Family Income: <$50k, Self-Report Health Status: Fair/Good

^a^Logistic regressions models controlled for sample weights; all models included adjusted for all covariates

^b^Total family income from all sources before taxes in 2008

### Underuse of care

Overall, 66 % of respondents felt that general underuse was a problem, 11 % had personally experienced underuse, and 8.5 % reported cost-related underuse. In bivariate analyses, minorities, less educationally or economically advantaged individuals and the uninsured had greater general concerns about underuse. Non-Hispanic Blacks were more likely than all Others to perceive general underuse as a problem, were more likely than Hispanics and Whites to report personal experiences of underuse, and to indicate that they had experienced cost-related underuse.

Race/ethnicity remained a significant predictor for just one dimension of underuse, in the multivariate regressions (Table 4). Blacks and Hispanics were more likely than Whites to report general concerns with underuse (AORs [95 % CIs], 3.07 [1.72 to 5.45], and 2.12 [1.24 to 3.61], respectively), but Others were less likely (AOR [95 % CI], 0.43 [0.23 to 0.80]). Race/ethnicity was not associated with personal experiences with underuse or cost-related underuse, after adjustment.

## Discussion

We examined race/ethnicity differences in Americans’ attitudes about and experiences with overuse and underuse of medical care. Our hypothesis that racial/ethnic minority respondents would be more likely to report both general concerns and personal experiences with underuse, was supported in the bivariate results and by the multivariate results for the general question about underuse, but not with regard to personal or cost-related experiences of underuse. Though we expected that Whites would perceive and experience more overuse, multivariate results indicated no race differences in general perceptions of overuse, with only Hispanics reporting fewer personal experiences of overuse than Whites. Conversely, both Blacks and Others were more likely to report cost-related overuse, while Others more often reported their doctors protecting them from overuse.

This study was limited by the age of the data, which was collected in 2009 and could be considered old. However, in the absence of other national surveys of attitudes and experiences about overuse and underuse in this era of great interest in the topic, these data still have significant value, particularly since deeply held attitudes are often enduring and difficult to change [14]. The fact that these data precede the introduction of national campaigns such as Choosing Wisely gives these data value as a baseline indicator about American attitudes, prior to the recent increase in attention to the issue of overuse. The data are also limited by the fact that they were derived from self-report, without objective assessments of actual care received or its appropriateness, or of conversations with doctors; however, the reliability of patient self-reports about their clinical experiences has been validated [15], and support the value of these data. Finally, the representativeness of the sample should be considered. When compared to results from the 2010 US Census [16], this study undersampled Hispanics, males, and the uninsured [9], but oversampled Whites, the elderly, the insured and individuals with poorer health. While 22 % had attended college and 42 % of the sample graduated college, this is roughly similar to the 23 % attending college and 38 % graduating college, as reported in national statistics from the American Community survey [17]. Thus, it may be especially notable that race/ethnicity differences were found, given the lower proportions of some minority groups, better health, and greater rates of insurance in the study sample.

Blacks’ and Hispanics’ reports of more general, but not personal or cost-related concerns about underuse might reflect respondents’ awareness of the societal problem of underuse, but an inability to discern when they themselves are not receiving needed and appropriate care. If so, the need to tailor programs, policies and educational campaigns designed to heighten patients’ understandings of appropriate vs. inappropriate care becomes increasingly salient. Perhaps these findings reflect generally high degrees of trust in one’s own personal doctor, versus the medical system as a whole [18], or patients’ inability to critically evaluate their doctors’ recommendations [19, 20]. Educational campaigns, such as Choosing Wisely — begun in 2012 to raise Americans’ consciousness about issues of overuse [21] — aim to foster patients’ ability to more critically evaluate physician recommendations, and may help educate patients lack of awareness of personal over- and underuse. Similarly, insurers’ provision of health information, patient navigators and other programs to help patients navigate the health system might need to be tailored, or at least made sensitive, to the likelihood that patients from varying groups bring with them widely varying attitudes and experiences about the system.

## Conclusions

These findings indicate that while many Americans feel that over- and underuse of medical care are important problems, some of these perceptions vary significantly by race and ethnicity, suggesting significant racial/ethnic gaps in perceptions and experiences with medical care among the American public. Some of these concerns could potentially be ameliorated in clinical settings through better communication or health information with patients, especially regarding the appropriateness of treatment recommendations. These variations in Americans’ perceptions are also important to recognize by those leading educational campaigns, such as the ABIM Foundation’s Choosing Wisely campaign, or patient education efforts by Consumer Reports, to educate Americans about the issues regarding overuse. The dynamics are also salient for disparities researchers and others addressing issues of underuse. Insofar as “one size does not fit all”, Americans’ attitudes about overuse and underuse of medical care varies with personal characteristics and group membership. Educational materials and informational campaigns may need tailored messages and messaging strategies for varying patient population groups, and efforts to enhance physicians’ skills in help patients choose wisely [22] need to ensure that physicians are equipped and skilled in providing patients of all backgrounds with the information they need to ultimately improve the equity and quality of care for all.

## Abbreviations

NPR: National Public Radio
KFF: Kaiser Family Foundation
HSPH: Harvard School of Public Health
